# Assisted evaluation of aniline's *in silico* toxicity using artificial intelligence and its simultaneous determination as a toxic impurity with widely used cardiovascular drugs using a green micellar chromatographic method

**DOI:** 10.1039/d5ra09502f

**Published:** 2026-02-18

**Authors:** Alaa Ahmed Mostafa, Soad S. Abd El-Hay, Youstina Mekhail Metias, Mohamed Adel Said, Michael Gamal Fawzy

**Affiliations:** a Pharmaceutical Chemistry Department, Faculty of Pharmacy, Egyptian Russian University Badr City Cairo 11829 Egypt alaaazzam30@gmail.com; b Pharmaceutical Analytical Chemistry Department, Faculty of Pharmacy, Zagazig University Zagazig 44519 Egypt

## Abstract

The safety of pharmaceutical products is critically influenced by the presence of impurities and degradation products. Aniline (ANN) is a very toxic degradation product of atorvastatin (ATN), which can cause life-threatening diseases such as methemoglobinemia. For the first time, a comprehensive artificial intelligence study using molecular docking was applied to assess the ANN-induced methemoglobinemia by simulating different binding energies in different pockets of cytochrome P450 (CYP 1A2), revealing the most suitable position leading to toxicity. Moreover, environmental concerns have become increasingly important due to the toxic effects of the excessive use of organic solvents in chromatographic separation systems. Accordingly, greener surfactant systems comprising sodium dodecyl sulfate (SDS) and polyoxyethylene-23-lauryl ether (Brij-35) were employed as safer alternatives were used for the quantitation of ANN in the presence of salicylic acid as an aspirin impurity alongside four widely used cardiovascular drugs of aspirin (APN), atenolol (AEN), atorvastatin calcium (ATN), and losartan potassium (LSN) in pure form and pharmaceuticals. The method validation was done according to the International Conference for Harmonisation (ICH) guidelines with linearity ranges of (10–200), (5–140), (5–100), (5–130), (0.5–40), and (0.5–30) µg mL^−1^ for APN, AEN, ATN, LSN, SAA, and ANN, respectively, and the results obtained were highly accurate. The greenness of the proposed method was ascertained using the green analytical procedure index: score (0.80), blue applicability grade index: score (0.85), and the Analytical GREEnness calculator. A statistical comparison between the results of our study and the reported method showed no significant difference in precision or accuracy.

## Abstract 2

An eco-friendly micellar HPLC method, integrated with an AI-assisted docking approach, enables the simultaneous determination of aniline and salicylic acid impurities with four cardiovascular drugs, to predict aniline toxicity.

## Graphical abstract

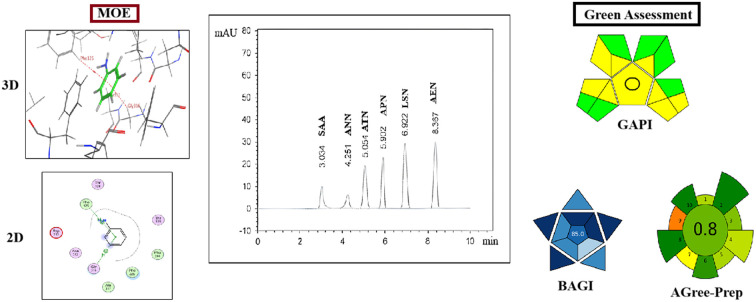

## 1 Introduction

Cardiovascular diseases, particularly hypertension and hypertriglyceridemia, are two of the most prevalent and lethal non-communicable diseases.^1^ Hypertriglyceridemia, characterized by high levels of triglycerides in the blood,^3^ is usually one of the main causes of hypertension, characterized by persistent elevation of blood pressure.^2^ Both conditions are often linked to multiple factors, such as poor lifestyle, and can significantly increase the risk of morbidity and mortality.^4^ Annually, cardiovascular diseases, particularly those driven by hypertension and hypertriglyceridemia, result in over 6 million deaths worldwide.^5^

Managing patients with both hypertension and hypertriglyceridemia often requires a combination of lifestyle modifications and pharmacological interventions.^6^ Lifestyle changes can include regular physical activity, a diet rich in potassium and low in sodium, and moderate or no alcohol consumption.^7^

Nearly 95% of hypertension cases that are complicated with hypertriglyceridemia require pharmacological treatment, often involving a combination of drugs with diverse mechanisms of action.^2^ To improve treatment adherence in these complex cases, fixed-dose combinations of two drugs in a single tablet can be beneficial.^3^

**Fig. 1:**
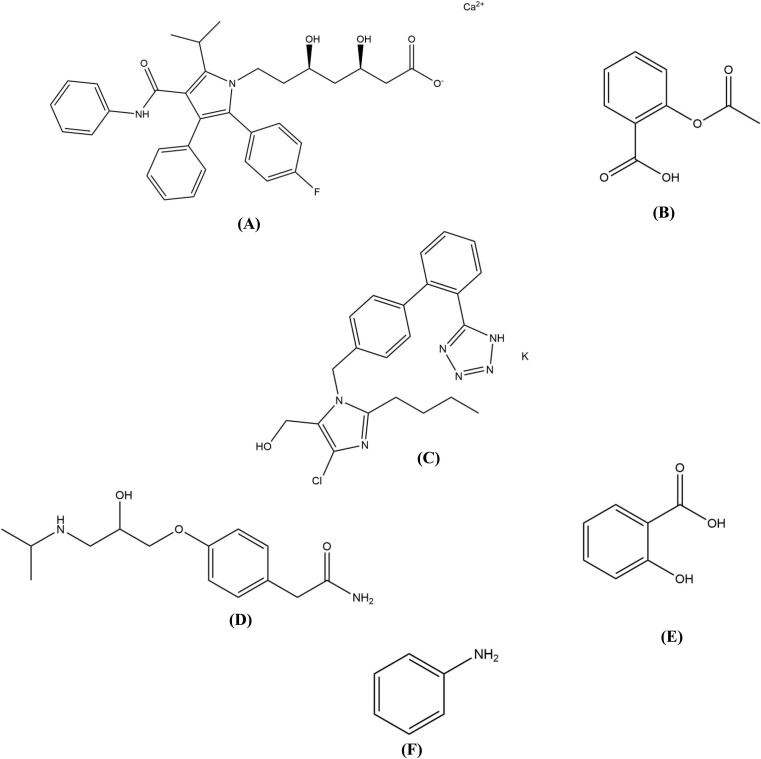
Chemical structures of (A) atorvastatin calcium, (B) aspirin, (C) losartan potassium, (D) atenolol, (E) salicylic acid, and (F) aniline.

Atorvastatin calcium (ATN) (Fig. 1A)^4,5^ works by competitively inhibiting 3-hydroxy-3-methylglutaryl-coenzyme A reductase,^6^ thereby impeding mevalonate synthesis and subsequently reducing hepatic cholesterol production. This reduction in cholesterol levels triggers an increase in LDL receptor expression in liver cells, leading to enhanced LDL uptake from the bloodstream.^7^

Aspirin (APN) (Fig. 1B)^4,5^ is an anti-inflammatory drug that inhibits the synthesis of prostaglandins through the deactivation of the cyclooxygenase enzyme pathway.^8^ APN was utilized in this combination to increase pain tolerance and maintain optimum blood flow.^9^

Losartan potassium (LSN) (Fig. 1C)^4,5^ is a selective and competitive blocker of angiotensin receptors, enabling a compensatory elevation of renin and angiotensin I levels.^10^ LSN effectively lowers blood pressure, reducing the risk of stroke and heart disease. LSN may also improve kidney function in patients with diabetes or other kidney issues.^11^

Atenolol (AEN) (Fig. 1D)^4,5^ is a selective inhibitor of β_1_ adrenergic receptors in vascular smooth muscles and the heart. This selective binding blocks the positive inotropic and chronotropic actions,^12^ allowing the heart to maintain a regular rhythm with minimum side effects.^13^ A quaternary combination of ATN, APN, LSN, and AEN, a combination with established medicinal value,^14^ along with appropriate lifestyle changes, can effectively treat complex cardiovascular conditions.^15^ These drugs offer different complementary non-interfering mechanisms of action with additional pain-relieving and anti-inflammatory effects. In addition, their minimal adverse effects ensure optimum management of even the most complicated conditions.

Salicylic acid (SAA)^5^ is a known major APN impurity listed in BP^14^ with a limit of 0.02% and aniline (ANN)^5^ is an approved ATN degradation product with a related structure,^16^ which were studied due to their potential toxicities (Fig. 1E and F). ANN can cause methemoglobinemia by inducing oxidation of the iron center in haemoglobin, leading to several blood complications.^17^ ANN toxicity was confirmed by simulation of its binding to CYP1A2.^18^

Micellar liquid chromatography (MLC) is a type of reversed-phase liquid chromatography (RPLC) that employs a mobile phase consisting of an aqueous surfactant solution at concentrations exceeding its CMC. It is a good separation technique that uses environmentally safe and eco-friendly reagents to meet the standards of green chemistry.^19^ The versatility of the technique stems from the diverse spectrum of interactions that exist between the aqueous phase, stationary phase, eluted solutes, and micelles. The stationary phase in MLC is altered by the adsorption of surfactant monomers, creating an open micelle-like structure. Thus, the silanophilic interactions are diminished. When non-ionic surfactants are used, the polarity of the stationary phase is modified. Ionic surfactants, on the other hand, cause a net positive or negative charge at the surface of the stationary phase, with significant outcomes.^20^ Sodium dodecyl sulfate (SDS), an anionic surfactant, is widely used in MLC to separate positively charged chemicals by altering the stationary phase. A non-ionic biodegradable surfactant, Brij-35(polyoxyethylene-(23)-dodecyl ether), has also been recently employed. It lowers the polarity of the stationary phase, which decreases the retention of moderately polar compounds and enhances their separation without requiring excessive amounts of organic solvents.^21^ Therefore, our study employed MLC with Brij-35 and SDS combination to achieve rapid separation of the quaternary combination therapy without the need for highly hazardous organic modifiers.

Chromatographic methodologies were used for the estimation of cardiovascular drugs in their pharmaceutical formulations.^22–26^ As the safety of pharmaceutical products is critically influenced by the presence of impurities, degradation products like ANN, which is considered a toxic degradation product of ATN, can cause life-threatening diseases like methemoglobinemia. So, a comprehensive artificial intelligence study using both molecular docking and ADMET profiles was applied to assess the ANN-induced methemoglobinemia by simulating different binding energies in different pockets of cytochrome P450 (CYP1A2), revealing the most favourable position leading to toxicity. The safety of pharmaceutical products is critically influenced by the presence of impurities and degradation products.^27^

Artificial intelligence (AI) integrated into software tools plays a supportive yet powerful role in molecular docking by predicting the mechanism of binding between ANN and CYP, on which induction to CYP was applied and complementary ADME (absorption, distribution, metabolism, and excretion) were conducted to confirm toxicity. In our work, MOE and ADME analyses are AI-assisted, meaning that AI algorithms help improve accuracy and efficiency but do not fully replace human control or scientific interpretation.^28,29^ AI aids in predicting ligand–receptor binding affinities, analyzing large datasets for pharmacokinetic properties, and optimizing docking simulations. This assistance makes virtual screening faster, more precise, and cost-effective, while expert validation ensures reliability and scientific soundness in drug discovery.^30,31^

Despite the widespread use of ATN and its known degradation to ANN, there are no comprehensive studies combining *in silico* toxicity simulation with green analytical quantification of ANN and related impurity in multicomponent cardiovascular drug formulations. Furthermore, the toxicological behaviour of ANN at the molecular level, particularly its interaction with CYP1A2, has not been thoroughly investigated using artificial intelligence-driven approaches. In addition, most previously reported HPLC methods rely on large volumes of organic solvents and do not address the environmental impact or the simultaneous detection of impurities such as ANN and SAA.

Therefore, the proposed method was specifically designed for the simultaneous determination of the studied compounds in the presence of their related impurities and degradation products, differing in scope, analytical matrix, and objectives from previously published methods. Building on this framework, the innovative aspect of this study was to address existing gaps by integrating molecular docking and ADMET analyses to provide predictive insights into aniline-induced toxicity, alongside the development and validation of an eco-friendly micellar HPLC method for the simultaneous determination of ANN, SAA, and four commonly prescribed cardiovascular drugs in bulk materials and pharmaceutical dosage forms. The combined experimental–computational approach represents a key advantage, as it enables efficient toxicity screening, reduces reliance on extensive *in vivo* testing, and offers a green, cost-effective, and robust analytical solution suitable for routine quality control. This integrated strategy supports informed decision-making when selecting analytical methodologies based on regulatory, environmental, and practical considerations. As a limitation, the toxicity assessment relies on *in silico* prediction models, which serve as supportive, preliminary risk-assessment tools and do not replace definitive biological or clinical toxicity confirmation. Consequently, the computational findings should be interpreted as complementary to, rather than a substitute for, experimental toxicological evaluation.

## 2 Experimental

### 2.1 Instrumentation

An Agilent 1100 series apparatus (DE 43630820) was paired with an isocratic quaternary pump (model G1311A) and a Rheodyne autosampler injector connected to a Symmetry Kinetex C_18_ column (100 × 4.6 mm, 5 µm) from Phenomenex, USA for chromatographic separation. A variable wavelength UV detector (model G1314A) was utilized with a standard flow cell (10 mm and 1000 psi standard pressure) for analysis.

A Benchtop pH meter PB3001 (Trans-instruments, India) for precise pH measurements of the mobile phase and sonicator (model DC-80H) were also utilized in this study.

### 2.2 Software

The HPLC apparatus was operated, and data were collected using Agilent ChemStation^®^ software. Docking simulations were conducted using the molecular operating environment (MOE^®^, 2022). In the toxicity investigation, the ADMET web tool was investigated and used. Statistical analysis and data processing were done using Microsoft Excel^®^.

### 2.3 Materials, reagents, and pharmaceutical products

The pure active pharmaceutical ingredients, AEN, ATN, and LSN were obtained from EIPICO (Egypt) and APN was supplied by Marcyrl Pharmaceuticals, Egypt, with purities of 98.70, 100.00, and 99.90, and 99.92%, respectively.^24^ SAA and ANN were purchased from Loba Chemie Manufacturers, India. Sodium dihydrogen phosphate, sodium dodecyl sulfate (Sigma-Aldrich), Brij-35 (Alfa Aesar), methanol (Adwic, Egypt), and HPLC-grade water (Tedia) were used in the present study.

The following available tablets from the Egyptian markets were used for preparing various laboratory ratios: Ator^®^ (10 mg ATN/tablet) with BN 2404319 manufactured by EIPICO, Ezacard^®^ (75 mg APN/tablet) with BN AT240307 produced by multiapex Pharma, Amosar^®^ (50 mg LSN/tablet) with BN 230790 manufactured by AMOUN, and Atelol^®^ (50 mg AEN/tablet) with BN 230450 manufactured by Pharco Pharmaceuticals.

### 2.4 Chromatographic conditions

Chromatographic separation of the quaternary combination in the presence of SAA and ANN was performed using an isocratic elution of a mobile phase consisting of 90% (0.15 M SDS and 0.05 M Brij-35) aqueous solution adjusted to pH 5.0 with 0.05 M NaH_2_PO_4_: 10% 1-butanol. The mobile phase was pumped at a flow rate of 1.0 mL min^−1^ through an appropriate Kinetex C_18_ (100 mm × 4.6 mm × 5 µm) column maintained at 25 °C. The injection volume of each sample into the Kinetex column was 10 µL, and 230 nm was the selected wavelength. Optimal functioning is preserved by column conditioning with the eluent system 10 minutes before injection. The chromatographic apparatus was first cleaned with water and then rinsed with a water–butanol combination (1 : 1, v/v) for 15 minutes to eliminate the adsorbed surfactants from the stationary phase.

### 2.5 Preparation of standard solutions

Standard stock solutions of APN, AEN, ATN, and LSN (1000 µg mL^−1^) and a stock solution of SAA (1000 µg mL^−1^) were prepared by dissolving 100 mg of each compound in 40 mL of the mobile phase using sonication for 3 minutes. The final volumes of individual volumetric flasks were then completed to 100 mL with butanol. A stock solution of ANN (1000 µg mL^−1^) was also prepared by transferring an equivalent volume of ANN into a 100 mL volumetric flask, where each 1 mL of ANN was equivalent to 1.020 g and diluting to the final volume with the mobile phase. Afterward, standard working solutions of APN, AEN, ATN, and LSN (200 µg mL^−1^) and working solutions of SAA and ANN (100 µg mL^−1^) were prepared by further diluting each stock solution with the mobile phase.

### 2.6 Preparation of laboratory prepared mixtures

Accurate volumes were transferred from the standard working solutions of the drugs into a set of 10 mL volumetric flasks to prepare mixtures in various ratios, including a 10 : 75 : 50 : 50 ratio of ATN, APN, LSN, and AEN, respectively, which constitute a medicinally recommended pharmaceutical dosage form. SAA and ANN impurities were also included in the prepared mixtures. SAA impurity was added according to the BP threshold, while the ANN impurity was added based on a calculated ratio relative to its corresponding drug, ATN. The final volume of each prepared mixture was adjusted to 10 mL with a mobile phase.

### 2.7 Computational studies

#### 2.7.1 Aniline binding molecular docking simulation

AI supports the docking process through embedded data-driven scoring, regression-based affinity predictions, and pattern-recognition algorithms. These AI-assisted functions help rank the docking poses, estimate binding affinity, and interpret ligand–protein interactions, while the core calculations remain based on classical molecular mechanics. CYP1A2 is an enzyme belonging to the cytochrome P450 superfamily, a group of enzymes that play a key role in the metabolism of various substances within the body. Found primarily in the liver, CYP1A2 is responsible for metabolizing a wide range of drugs.

CYP1A2 is particularly notable for its role in activating procarcinogen substances that become carcinogenic after being metabolized by the enzyme. Its activity can be influenced by genetic factors, lifestyle factors like smoking, and exposure to certain chemicals such as ANN.^17^

The main compound under investigation (ANN) was installed in MOE in a 3-dimensional model view and its integrity was checked. Energy minimization was applied to ANN and charges were carefully monitored, based on the 2-dimensional depiction. The calculation of charges was automated by MOE. Finally, the ANN was stored as an MDB file to be used in the docking process and calculations.

##### 2.7.1.1 Preparation of the target protein structure

Crystalline configuration of (CYP1A2) was acquired from Protein Data Bank (https://www.rcsb.org) as [PDB id: 2HI4] (a computed complex structure 1A2) with a total weight of the structure of 57.18 kDa.^18^ Through trials, most of the atom types and their bonding were revised and mended utilizing the order of fixation in the MOE system. A number of steps were taken to enhance the final molecular docking simulation. First, the protonation step was utilized to enhance binding activity by the addition of hydrogen atoms, reflecting a stronger interaction between ANN and CYP1A2. Second, a confining ligand (CYP1A2) was applied to detect the binding site. Third, the removal of co-crystallized water molecules from the confined compound.

##### 2.7.1.2 Preparation of tested drug molecules

MOE was used to prepare a 3D model from ANN. This compound was subjected to energy minimization and automatic calculation of the partial charges. Finally, this prepared library was saved as an MDB file to be used in docking calculations with the Human Microsomal P450 1A2 enzyme.

#### 2.7.2 ADMET LAB 2.0 – drug-likeness and ADME studies

AI is widely applied in ADMET prediction to analyze large chemical datasets and identify patterns linking molecular structures to pharmacokinetic and toxicity outcomes. Machine-learning and deep-learning algorithms enable rapid estimation of absorption, distribution, metabolism, excretion, and potential toxic effects, supporting early-stage drug development and prioritization of compounds, while experimental validation remains essential. The ADMET LAB 2.0 platform (https://admetlab3.scbdd.com/) is a freely accessible web tool that combines the most relevant computational techniques to provide a global estimation of the pharmacokinetics profile of small molecules. Its methodologies were chosen by the web tool creators for their robustness, as well as their ease of interpretation, to allow effective translation into medicinal chemistry. Some of these approaches were updated by web tool designers utilizing open-source algorithms, while others were unmodified versions of the methods of the original authors. The molecular structure of ANN was uploaded to the ADMET LAB 2.0 web tool section using the simplified molecular-input line-entry specification (SMILES) nomenclature technique using Marvin sketch software 19.19, and then the result report was generated.

### 2.8 Procedure

#### 2.8.1 Linearity and construction of calibration curve

Different precise aliquots of the studied drugs (ATN, APN, LSN, and AEN) along with SAA and ANN impurities were transferred from their respective fresh working solutions into a series of 10 mL volumetric flasks. The final volumes were then completed with the mobile phase before auto-injection into the Kinetex column. The chromatographic measurements were performed for each concentration in triplicate (10 µL each) and analyzed under the proposed chromatographic conditions described in Section 2.4. The final achieved concentrations for the studied mixture were 5–100, 10–200, 5–130, 5–140, 0.5–40, and 0.5–30, respectively. The peak areas recorded at 230 nm against the corresponding concentrations (µg mL^−1^) were used to construct calibration curves for each drug and impurity, followed by linear regression analysis.

#### 2.8.2 Analysis of laboratory-prepared mixtures

Into a series of 10 mL volumetric flasks, different aliquots from the standard stock solutions of the aforesaid drugs (ATN, APN, LSN, and AEN) were transferred. The resulting laboratory-prepared mixtures containing a 10 : 75 : 50 : 50 ratio of these drugs with various aliquots of SAA (as an APN impurity) and ANN (as an ATN degradation product) were prepared. The developed chromatographic condition was applied to analyze the prepared mixtures.

#### 2.8.3 Pharmaceutical dosage form analysis

Ten tablets of each drug were accurately weighed and ground into a fine powder. An amount of powder equivalent to the average tablet weight was dissolved in 30 mL of the mobile phase, sonicated for 10 minutes, and filtered individually through a 0.45-micron syringe filter. The filtrate was transferred into a 100 mL volumetric flask and diluted to the final volume with the mobile phase. The prepared drug solution had final concentrations equivalent to 100, 750, 500, and 500 µg mL^−1^ for ATN, APN, LSN, and AEN, respectively, based on each labeled drug. Chromatographic analysis was performed as described in Section 2.8.1 using the standard addition approach, which entailed adding each drug's pure standard to the relevant mixture prior to carrying out the required chromatographic tests, and the validity was evaluated.

### 2.9 Method development and optimization

To optimize the separation of SAA and ANN in various laboratory-prepared mixtures containing ATN, APN, LSN, and AEN, multiple experiments were conducted to identify the optimal chromatographic conditions using the proposed micellar-HPLC method. These experiments involved varying factors such as wavelength, mobile phase composition and ratios, flow rate, and pH.

To achieve better chromatographic separation of the analytes on the selected column of C_18_, the trials were applied, testing several parameters, including: first, analysis of the combined mixture at various wavelengths (230, 250, and 285 nm) for the optimal wavelength for the simultaneous detection of all components in different concentration ratios. Second, the mobile phase composition, including micellar eluents of SDS (an anionic surfactant) in the presence of Brij-35 (a non-ionic surfactant) at different ratios, with the possibility to add an organic modifier, was tested.

After adjusting the mobile phase composition and ratio, it was necessary to determine the suitable pH value of the chosen mobile phase and a reasonable flow rate of its isocratic elution. In addition, the tested pH values (3–7) were prepared using 0.05 M phosphate buffer (NaH_2_PO_4_) in HPLC water, adjusted with phosphoric acid or NaOH. Finally, a range of flow rates from 0.75 to 1.5 mL min^−1^ was also investigated thoroughly to achieve a balance between a suitable total run time and good chromatographic separation.

### 2.10 Method validation

ICH guidelines^32^ were utilized to assess the validity of the proposed chromatographic method.

#### 2.10.1 Linearity and range

The linearity of the proposed method was studied utilizing eight different concentrations with different ranges for each drug and impurity. Plotting analyte concentrations against their related peak areas was utilized to construct calibration curves and for further statistical analysis. The linear regression equations and determination coefficients (*r*^2^) were then established.

#### 2.10.2 LOD and LOQ

LOD and LOQ were calculated as follows:LOD = 3.3 × SD/*S*LOQ = 10 × SD/*S*Here, SD is the standard deviation of the intercept and *S* is the slope of the calibration curve.

#### 2.10.3 Accuracy

Estimation of five distinct concentrations of APN (20, 60, 100, 140, and 190 µg mL^−1^), AEN (10, 35, 80, 110 and 130 µg mL^−1^), ATN (10, 30, 55, 80, and 95 µg mL^−1^), LSN (10, 30, 70, 90, and 110 µg mL^−1^), SAA (3, 7, 18, 22, and 27 µg mL^−1^), and ANN (3, 7, 18, 22, and 27 µg mL^−1^) was conducted three times to assess the accuracy of the proposed HPLC method.

#### 2.10.4 Precision

Three different concentrations of APN (20, 100, and 190 µg mL^−1^), AEN (10, 80, and 110 µg mL^−1^), ATN (10, 55, and 95 µg mL^−1^), LSN (10, 70, and 110 µg mL^−1^), SAA (3, 18, and 27 µg mL^−1^), and ANN (3, 18, and 27 µg mL^−1^) were analyzed three times in one day and in three successive days to investigate intraday and interday precision, respectively.

#### 2.10.5 Robustness

By studying the effect of small modifications on the suggested chromatographic conditions, robustness was evaluated. The composition of the mobile phase was maintained, while the temperature, pH value, and flow rate were changed by ± 2 °C, ± 0.1, and ± 0.1, respectively.

#### 2.10.6 Specificity

Several laboratory-prepared solutions containing the Starpill^®^ ratio (75 : 50 : 10 : 50) in the presence of different concentrations of SAA and ANN impurities (0.5, 5, 10, 20, and 30 µg mL^−1^) were evaluated to investigate the selectivity of the proposed method.

#### 2.10.7 System suitability

USP guidelines^33^ were utilized to check the system suitability parameters of the proposed HPLC method. Each parameter was calculated and compared to the USP reference values.

### 2.11 Pharmaceutical application

Utilizing a standard addition approach by the addition of different ratios of pure drugs to assess the accuracy of the proposed HPLC method for the estimation of ATN, APN, LSN, and AEN in their combined dosage form was applied.

### 2.12 Statistical analysis

Statistical evaluation and comparison of the proposed HPLC method in terms of accuracy and precision with the reported HPLC method^24^ was applied.

### 2.13 Computational studies

#### 2.13.1 Aniline binding molecular docking simulation

MOE (version 2022) was utilized to perform molecular docking simulation through the preparation of both protein and ligands, molecular docking, and evaluation of ligand–protein interactions through visualization of poses and scoring functions. The docking process was performed *via* the Amber10 protocol using docking placement: triangular matcher, rescoring: London dG, forcefield and refinement: affinity dG.

MOE 2022 was used for molecular docking simulation to comprehend the unique recognition properties of ANN with CYP1A2. To determine the binding characteristics and identify the connections between the ANN's structural properties and affinity profile at the biggest pocket of the protein crystal structure, docking studies were conducted.

#### 2.13.2 ADMET LAB 2.0 – drug-likeness and ADME studies

The ADMET properties of ANN were predicted using the ADMET LAB 2.0 web tool (https://admetlab3.scbdd.com/). The compound demonstrated excellent human intestinal absorption (HIA), and its compliance with Lipinski, Pfizer, and GSK rules further supported its drug-likeness. Additionally, ANN was able to cross the blood–brain barrier (BBB).

### 2.14 Chromatographic method sustainability assessment

A part of the evaluation of the developed micellar HPLC method is to assess its greenness sustainability since the developed method utilized a mobile phase composed of an aqueous surfactant solution (SDS and Brij-35) and a small volume of organic modifiers, such as *n*-butanol (which is greener than other solvents, *e.g.*, methanol).^35^ The micellar system was evaluated using different assessment methods, such as:

1. Green analytical procedure index (GAPI)^36^ is a green assessment tool that describes 12 green metrics in the shape of 15 geometric parts. GAPI was utilized to establish a thorough green evaluation of the micellar HPLC method, including all in-process steps from the sample, SDS, and Brij-35 preparations to waste disposal. The environmental impact of the employed device, including mobile phase consumption and potential harm, was also studied. According to the color-coding system set by the software provider, each color code indicates a different effect on the environment as follows: green for low, yellow for medium, and red for high impact.

2. Blue applicability grade index (BAGI),^37^ this green assessment tool consists of various geometrical shapes that assess 10 concepts of greenness. Among the assessed concepts is the quantitative and confirmatory nature of the analysis, the sample throughput of 5–10 samples per hour, and the use of simple, eco-friendly, highly recyclable reagents. Each of these concepts, along with other parameters, is assigned a score on a scale of 2.5 to 10 and a different blue color brightness.

3. Analytical GREEnness calculator (AGREE)^38^ is an updated system designed to assess the degree of greenness of any analytical methods. It depends mainly on a 10-section scale, and each takes a different or similar color based on its greenness level, calculated according to the basic principles of green chemistry. Assigning a score of 0 to 1 to each section with apparent colors is the main core of AGREE-prep. To encourage more environmentally friendly and secure practices, the calculator primarily evaluates different parameters, including the amount of reagent toxicity, generated waste, energy requirements, number of processes, miniaturization, and automation.

## 3 Results and discussion

### 3.1 Method development and optimization

After analysis of the combined mixture at various wavelengths (230, 250, and 285 nm), it was revealed that 230 nm was the optimal wavelength for the simultaneous detection of all components in different concentration ratios. It offered sufficient sensitivity for the determination of the mixture in the presence of lower concentrations of the studied impurities, ANN and SAA, with consistent and well-defined peak shapes compared to higher wavelengths, which showed inferior chromatographic performance.

Also, it was found that micellar eluents of SDS in the presence of Brij-35 showed greater elution strength due to their capability of eluting all mixture components completely. However, given the numerous advantages of micellar eluent systems, such as their high elution strength, safe disposability, and favorable ecological profile, the use of a small proportion of a green organic modifier was a necessity for the developed method. The pure micellar mobile phase exhibited unpredictable retention time with significant tailing for some analytes, and it also affected column efficiency. Hence, the introduction of 1-butanol to the eluent system in a lower concentration (10%) as a greener organic modifier, rather than acetonitrile or methanol, was sufficient for significant enhancement of the chromatographic performance within a reasonable runtime.

**Fig. 2:**
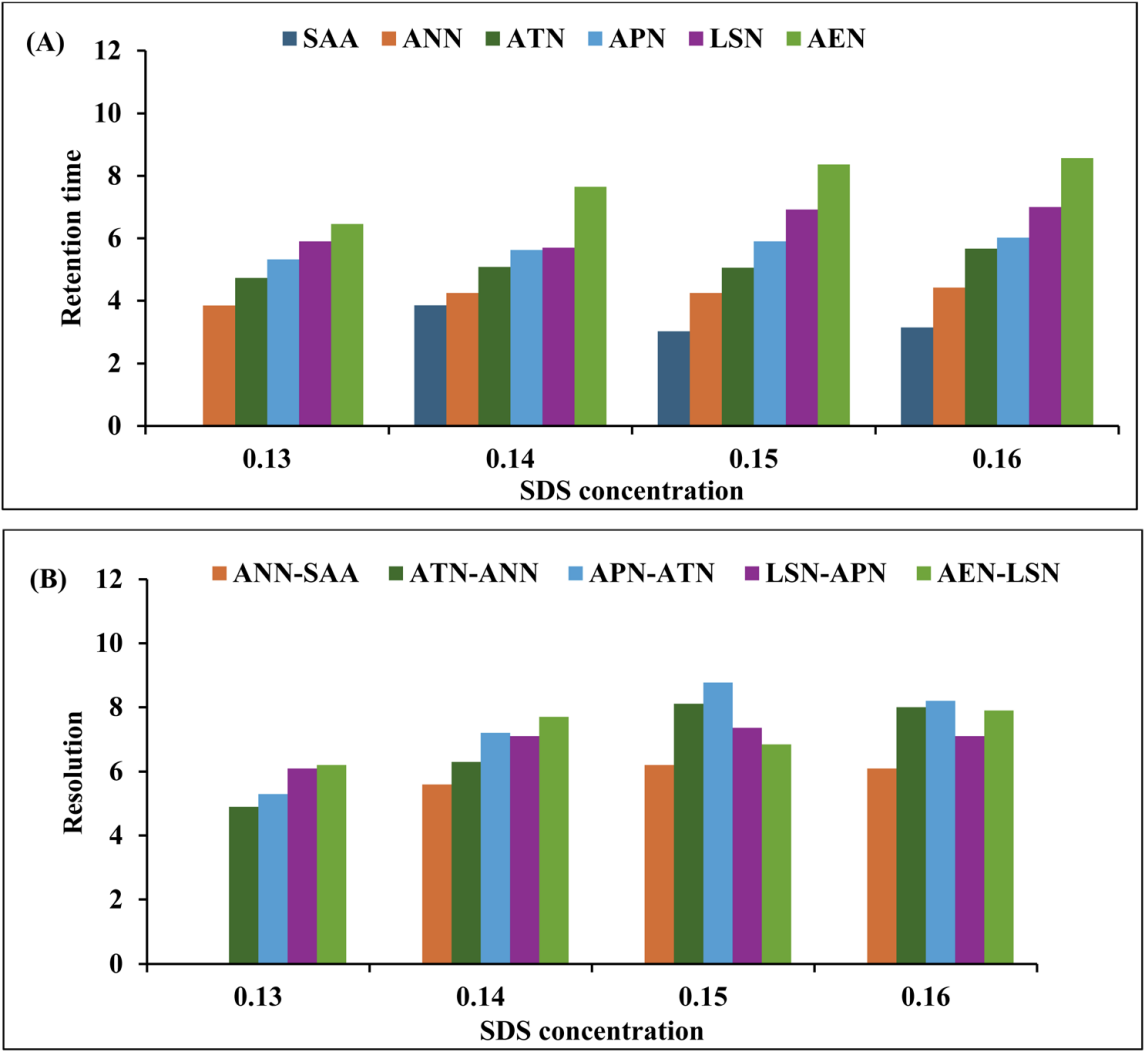
The impact of the SDS concentrations on the (A) retention time and (B) resolution of the studied mixture with a constant Brij-35 concentration.

**Fig. 3:**
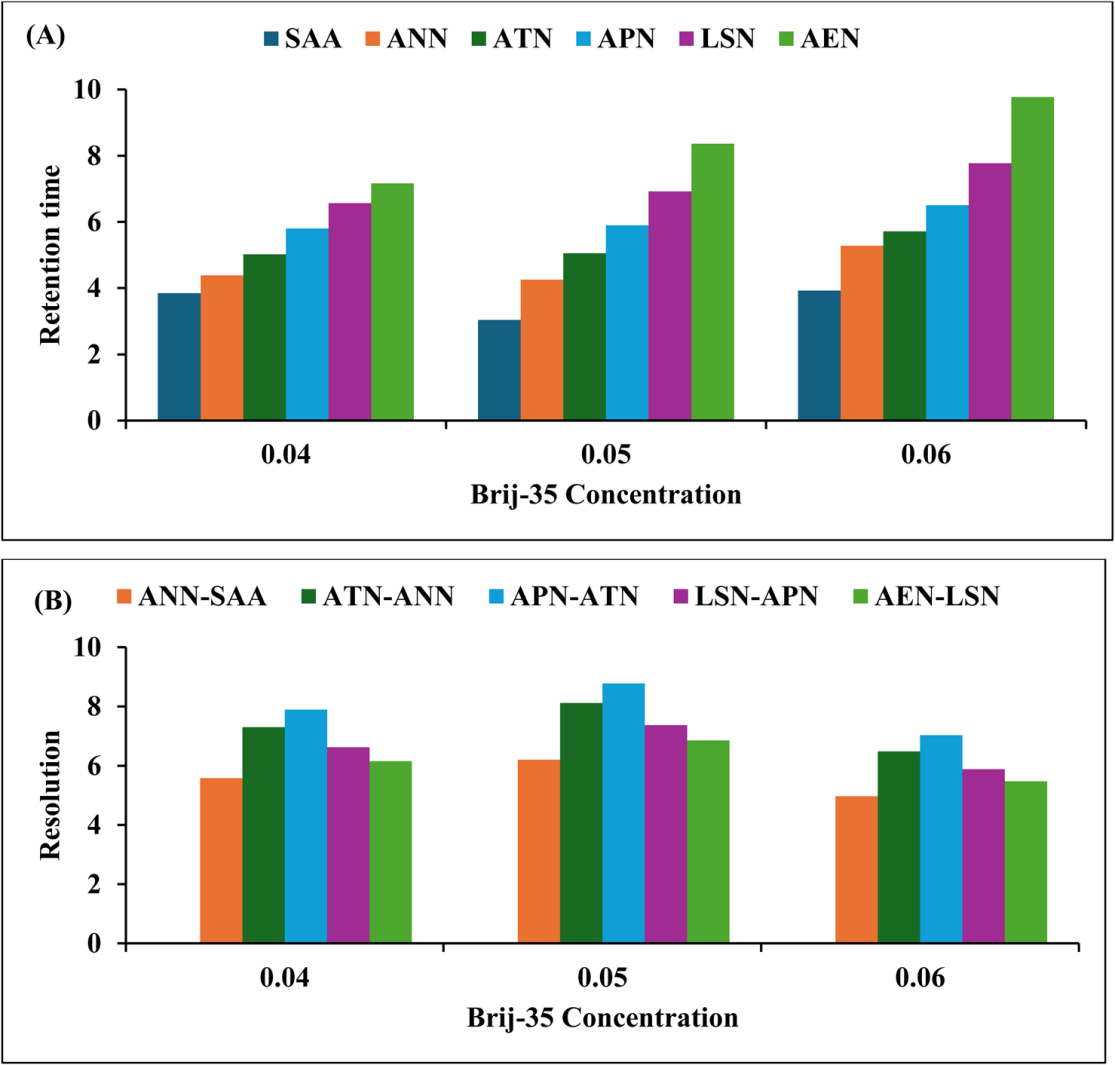
The impact of the Brij-35 concentrations on the (A) retention time and (B) resolution of the studied mixture with a constant SDS concentration.

Several trials employing SDS and Brij-35 at different concentrations of each while maintaining the other at a constant concentration were also conducted, and their effects on the retention times and resolutions of all analytes were monitored, as depicted in Fig. 2 and 3, respectively. For SDS, the concentrations lower than 0.15 M resulted in the peak tailing of certain drugs and affected the retention time greatly as shown in Fig. 2A, where the overlapped and least resolved peaks of APN and LSN were observed upon using 0.13 and 0.14 M of SDS as shown in Fig. 2B. Also, various concentrations of Brij-35 were studied to evaluate the most appropriate concentration. Concentrations below 0.05 M exhibited shorter retention times (Fig. 3A), whereas higher concentrations prolonged the retention times of certain analytes, such as AEN. In addition, Brij-35 concentration had a great impact on peak resolution and symmetry (Fig. 3B). Accordingly, the mobile phase composition was adjusted to 90% (0.15 M SDS and 0.05 M Brij-35) and 10% 1-butanol after extensive optimization of both the 1-butanol: micellar system ratio and the concentrations of SDS and Brij-35.

Among the tested pH values (3–7), pH 5 was the most suitable buffer for separating the studied mixture of six substances with different p*K*_a_ values. The acidic analytes, SAA and APN, at pH 5 are partially ionized while still maintaining sufficient hydrophobicity, resulting in improved retention and well-defined peak shapes. At the same time, the basic drugs, ANN, AEN, and LSN, remain adequately protonated, reducing excessive interaction, minimizing peak tailing and enhancing symmetry. Consequently, this intermediate pH 5 offers the most harmonized ionization profile for both acidic and basic components. It yields superior chromatographic performance with optimal resolution and consistent retention behavior for all investigated drugs across the mixture compared to highly acidic or basic pH values.

During flow rate investigation, it was found that lower flow rates prolonged the separation process, affecting peak symmetry, while flow rates above 1 mL min^−1^ resulted in peak interference, especially between SAA and ANN, in addition to an undesirable increase in column pressure. Therefore, the suitable flow rate of 1.0 mL min^−1^ was chosen as it successfully exhibited the necessary separation quality in an efficient timeframe within 8.5 minutes without placing excessive strain on the column pressure.

**Fig. 4:**
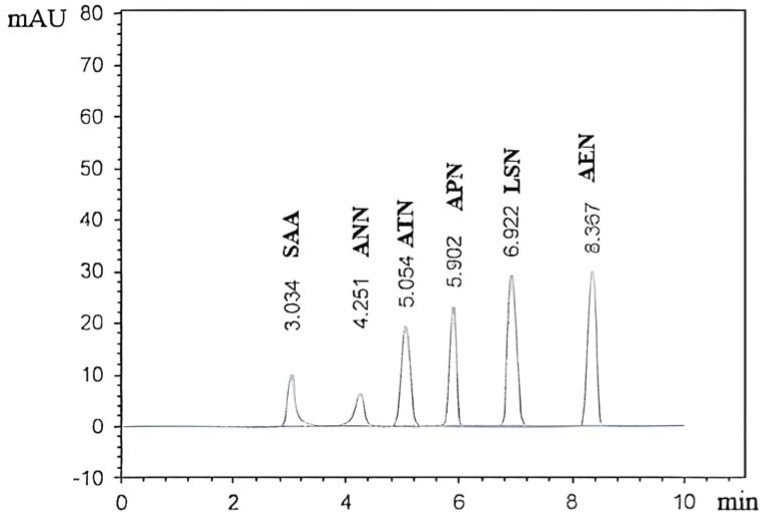
Micellar-HPLC chromatogram of SAA (10 µg mL^−1^), ANN (10 µg mL^−1^), ATN (10 µg mL^−1^), ARN (75 µg mL^−1^), LSN (50 µg mL^−1^), and AEN (50 µg mL^−1^) in their pure forms.

Finally, Fig. 4 was obtained utilizing the chromatographic conditions described in section (2.4), which includes: isocratic flow system with a ratio of 90% (0.15 M SDS and 0.05 M Brij-35) aqueous solution adjusted to pH 5.0 with 0.05 M sodium dihydrogen phosphate buffer: 10% 1-butanol was found to be the optimal for the mixture separation on a Kinetex C18 column maintained at 30 °C.

Setting the optimized chromatographic conditions was followed by comparison with the scientific literature, which revealed that the proposed method was specifically designed for the simultaneous determination of the studied compounds in the presence of their related impurities/degradation products and that it differs in scope, matrix, and analytical objectives from previously published methods. The relevant literature revealed the novelty, improved selectivity, greener solvent usage, and enhanced analytical performance of the proposed method compared to the reported approach. In addition, the present study incorporates computational toxicity assessment tools, including molecular docking and ADMET prediction models, to provide artificial intelligence–assisted, predictive insight into aniline-induced toxicity. This AI-supported approach complements the experimental chromatographic work by enabling preliminary toxicity screening and informed risk evaluation, which is not addressed in conventional chromatographic methods reported in the literature. The integration of green analytical chemistry with AI-based computational analysis, therefore, represents a distinctive and innovative contribution beyond previously published methods.

### 3.2 Method validation

ICH guidelines^32^ were utilized to assess the validity of the proposed chromatographic method.

#### 3.2.1 Linearity and range

The analyte concentrations were plotted against their related peak areas to construct the calibration curves. The linear regression equations and determination coefficients (*r*^2^) demonstrated exceptional linearity indicated by higher (*r*^2^) values of 0.9999, as shown in Table 1.

**Table 1:** Assay and validation parameters of the developed micellar chromatographic methodology

Parameters	HPLC					
	SAA	ANN	ATN	APN	LSN	AEN
Linearity range (µg mL^−1^)	0.5–40	0.5–30	5–100	10–200	5–130	5–140
LOD (µg mL^−1^)	0.13	0.06	1.63	2.46	0.48	0.44
LOQ (µg mL^−1^)	0.40	0.19	4.94	7.45	1.48	1.33
The determination coefficient (*r*^2^)	0.9999	0.9999	0.9999	0.9999	0.9999	0.9999
Slope	9.5828	4.5559	5.9357	6.9191	6.3944	6.5328
SE of slope	0.0199	0.0051	0.0156	0.0380	0.0116	0.0095
Intercept	−0.8626	−0.0294	−1.7606	−4.0579	−2.3124	−3.1444
SE of intercept	0.1840	0.08567	1.0334	5.1526	0.9251	0.8712
Intraday precision^a^	0.492	0.963	0.489	0.920	0.092	0.572
Interday precision^a^	0.762	0.287	0.557	0.384	0.359	0.586
Accuracy (mean ± % SD)	99.93 ± 1.125	100.20 ± 0.080	99.98 ± 0.354	99.82 ± 1.387	100.01 ± 0.515	100.09 ± 0.375

a Mean of three concentrations in triplicate within the same day and in three successive days.

#### 3.2.2 LOD and LOQ

The LOD and LOQ results illustrated in Table 1 showed low values of LOD and LOQ, proving the high sensitivity of the proposed HPLC method.

#### 3.2.3 Accuracy

By examining five distinct concentrations of each three times the excellent accuracy results (Table 1) were indicated by the means of % Recovery.

#### 3.2.4 Precision

Three different concentrations of APN, AEN, ATN, LSN, SAA, and ANN were analyzed three times in one day and on three successive days. Superior precision results were obtained by examining %RSD values, which were lower than 2% (Table 1).

#### 3.2.5 Robustness

Studying the effect of small modifications to the suggested chromatographic conditions revealed that the robustness of the devised HPLC technique did not show any significant differences in findings, regarding % Recovery and RSD (Table 2).

**Table 2:** Assessment of the robustness of the developed micellar chromatographic methodology

Parameters variation		SAA	ANN	ATN	APN	LSN	AEN
		(10 µg mL^−1^)	(10 µg mL^−1^)	(10 µg mL^−1^)	(75 µg mL^−1^)	(50 µg mL^−1^)	(50 µg mL^−1^)
		% Recovery					
Temperature	38 °C	99.95	99.81	100.36	99.63	101.11	100.57
	40 °C	100.26	100.52	101.21	99.69	101.50	100.74
	42 °C	100.45	99.95	99.88	99.30	101.08	101.15
	RSD	0.253	0.378	0.672	0.210	0.227	0.296
pH	4.9	99.60	99.69	99.68	99.39	100.60	100.78
	5	99.53	100.16	99.98	99.59	100.13	101.16
	5.1	100.08	101.38	100.23	100.02	99.86	100.78
	RSD	0.305	0.870	0.275	0.322	0.376	0.219
Flow rate	0.90	100.86	101.67	101.03	100.45	101.18	101.39
	1.0	100.21	100.37	100.86	99.50	100.53	100.58
	1.1	100.70	100.03	99.59	99.25	100.88	100.09
	RSD	0.338	0.859	0.782	0.637	0.319	0.651

#### 3.2.6 Specificity

After analysis of the laboratory prepared in (Table 3). It was confirmed that the optimum separation of the studied mixture with excellent resolution and minimum tailing of their peaks in the presence of each other and the studied impurities as demonstrated in Fig. 4. The specificity was also ascertained by assay of the analytes in their tablet pharmaceutical formulation, where the excipients did not exhibit any interferences (Table 4).

**Table 3:** Analysis of laboratory-prepared mixtures incorporated with different concentrations of SAA and ANN by the developed chromatographic methodology

	Taken (µg mL^−1^)		% Recovery					
	SAA	ANN	APN^a^ (75 µg mL^−1^)	AEN^a^ (50 µg mL^−1^)	ATN^a^ (10 µg mL^−1^)	LSN^a^ (50 µg mL^−1^)	SAA	ANN
	0.5	0.5	99.61	99.96	99.38	101.51	101.01	100.25
	5	5	98.14	99.99	100.41	100.58	100.62	100.99
	10	10	98.47	100.41	100.97	100.94	100.67	101.14
	20	20	98.30	100.68	98.74	100.63	100.71	100.79
	30	30	98.48	99.83	100.31	100.45	100.55	100.54
Mean			98.60	100.12	99.96	100.82	100.71	100.74
SD			0.579	0.413	0.891	0.427	0.069	0.256
% RSD			0.588	0.413	0.891	0.424	0.069	0.255

a Determining APN, AEN, ATN, and LSN in a medicinally recommended ratio of 75 : 50 : 10 : 50, respectively, in the presence of varying concentrations of SAA and ANN impurities.

**Table 4:** Application of the standard addition technique for the chromatographic analysis of the studied drugs in synthetic Starpill^®^ tablets

Micellar-HPLC															
ATN				APN				LSN				AEN			
Taken (µg mL^−1^)		% Recovery		Taken (µg mL^−1^)		% Recovery		Taken (µg mL^−1^)		% Recovery		Taken (µg mL^−1^)	% Recovery	Taken (µg mL^−1^)	% Recovery
Tablet	Added	Tablet	Added	Tablet	Added	Tablet	Added	Tablet	Added	Tablet	Added	Tablet	Added	Tablet	Added
	5		99.63		25		101.86		25		98.97		25		98.71
10	10	100.19	100.97	75	75	99.08	99.92	50	50	100.19	100.84	50	50	99.94	100.40
	20		99.88		100		99.66		75		100.35		75		100.03
Mean		100.16				100.48				100.05					99.71
SD		0.715				1.201				0.971					0.892
% RSD		0.714				1.195				0.971					0.894

#### 3.2.7 System suitability

As illustrated in Table 5, all the assessed parameters (retention factor (*K*), selectivity factor (*α*′), symmetry factor (*T*), theoretical plates (*N*), and peak resolution (*R*_s_)) complied with USP guidelines.

**Table 5:** System suitability of chromatographic quantification of SAA, ANN, ATN, APN, LSN, and AEN in their combined dosage form

Parameters	SAA	ANN	ATN	APN	LSN	AEN	Recommended value^33^
Retention time (*t*_R_) (min)	3.034	4.251	5.054	5.902	6.922	8.367	
Retention factor (*K*)	1.02	1.83	2.37	2.94	3.62	4.58	1–10
Resolution (*R*_s_)	—	6.21	8.11	8.77	7.36	6.85	≥2
Symmetry factor (*T*)	0.91	1.09	0.88	0.90	1.09	0.86	<1.5
Theoretical plate (*N*)	4226	4899	6333	7322	6698	5898	>2000
Selectivity factor (*α*′)	—	7.12	7.34	7.62	6.98	7.14	>1

### 3.3 Pharmaceutical application

As depicted in Table 4, the high recoveries of the added standards confirmed the accuracy and efficiency of the proposed method. The obtained recoveries demonstrate the method's accuracy and suitability for routine quality control, while the low %RSD values confirm acceptable precision in the analysis of commercial formulations. The absence of significant interference from common excipients further confirms the selectivity and robustness of the method when applied to real pharmaceutical matrices. Collectively, these findings verify that the proposed method is reliable for the simultaneous determination of the studied compounds in pharmaceutical dosage forms and is consistent with validated analytical performance criteria.

### 3.4 Statistical analysis

The analysis revealed no variations between the developed and reported methods, which were confirmed by the *t*-test and *F*-ratio values, as illustrated in Table 6.

**Table 6:** Statistical comparison of the results obtained by applying the developed and reported^24^ chromatographic methods

Form	Statistical parameters	Reported method^b^^24^				Micellar-HPLC			
		ATN	APN	LSN	AEN	ATN	APN	LSN	AEN
Pharmaceutical dosage form	Mean	99.92	99.62	100.10	99.49	100.19	99.08	100.19	99.94
	SD	1.016	0.780	0.562	0.521	1.124	0.414	0.485	0.358
	N	6	6	6	6	6	6	6	6
	Variance	1.040	0.608	0.314	0.270	1.254	0.168	0.240	0.130
	*t*-test (2.23)^a^	—	—	—	—	0.44	1.50	0.30	1.74
	*F*-ratio (5.05)^a^	—	—	—	—	1.21	3.62	1.31	2.09

a The tabulated values of the *t*-test and *F*-ratio.

b The reported HPLC method for the detection of ATN, APN, LSN, and AEN at 236 nm using a mobile phase of acetonitrile: 0.02 M potassium dihydrogen phosphate buffer of pH 3.4 (70 : 30, v/v).

This study establishes a comprehensive and regulatory-compliant analytical framework that delivers high sensitivity, robust linearity across the investigated concentration ranges, and excellent precision and accuracy, as evidenced by validation metrics that consistently meet accepted international guidelines with lower or similar LOD and LOQ values. In addition to achieving analytical performance comparable to or exceeding that of previously reported methods, the proposed approach extends selectivity by enabling the simultaneous quantification of active pharmaceutical ingredients in the presence of related impurities and degradation products. This capability addresses a critical limitation of many existing API-centered methodologies and directly enhances the methodological rigor and practical relevance of the research proposal. Collectively, these findings reinforce the scientific justification for the proposed work, support its applicability in routine quality control, stability studies, and regulatory submissions, and underscore its potential to improve the reliability, efficiency, and regulatory alignment of pharmaceutical analytical workflows.

### 3.5 Computational studies

#### 3.5.1 Aniline binding molecular docking simulation

To compare the receptor affinity and binding mechanism of ANN towards CYP 1A2, many metrics are considered. The amount of amino acids involved, the sorts of bonds they form, and the length of the bonds are these factors (Table 7).

**Table 7:** Strength of the binding mode of CYP 1A2 and ANN

Bond type	Bond strength (kcal mol^−1^)	Amino acid in CYP1A2
Hydrogen bond	1–5	Phe 125
Covalent bond	1	Gly 316
Electrostatic interaction	5–10	Asp 313
rseq	1	
mseq	1	
*S*	−4.1921	

##### 3.5.1.1 Validation of docking

The binding site in CYP 1A2 was a large pocket that allowed different binding conformations with ANN, resulting in the formation of many different bonds with different lengths and energies, which suggests that ANN may bind with different alignments within the CYP1A2 pocket. Unlike (Table 7) which showed the final most stable binding activity (Table 8), showed that other possible bonds may formed during movement of ANN inside pocket as π–π stacking between the ANN aromatic ring and the side chain of amino acids as phenylalanine (Phe) and tyrosine (Tyr), hydrophobic interaction utilizing the nonpolar residue of leucine (Leu), Isoleucine (Ile), and Valine (Val). Other possible interactions may occur, *e.g.*, van der Waals interactions that occur by proximity. This weak interaction is necessary to stabilize the formed complex.

**Table 8:** Strength of other possible binding modes of CYP 1A2 and ANN

Bond type	Bond strength (kcal mol^−1^)	Amino acid in CYP1A2
Hydrogen bond	1–5	Serine (Ser), threonine (Thr)
Pi–Pi stacking	2–10	Phenylalanine (Phe), tyrosine (Tyr)
Hydrophobic interaction	0.5–2	Leucine (Leu), isoleucine (Ile), valine (Val)
van der Waals	<1	Various amino acids
Ionic interaction (if applicable)	5–10	Glutamic acid (Glu), aspartic acid (Asp)

**Fig. 5:**
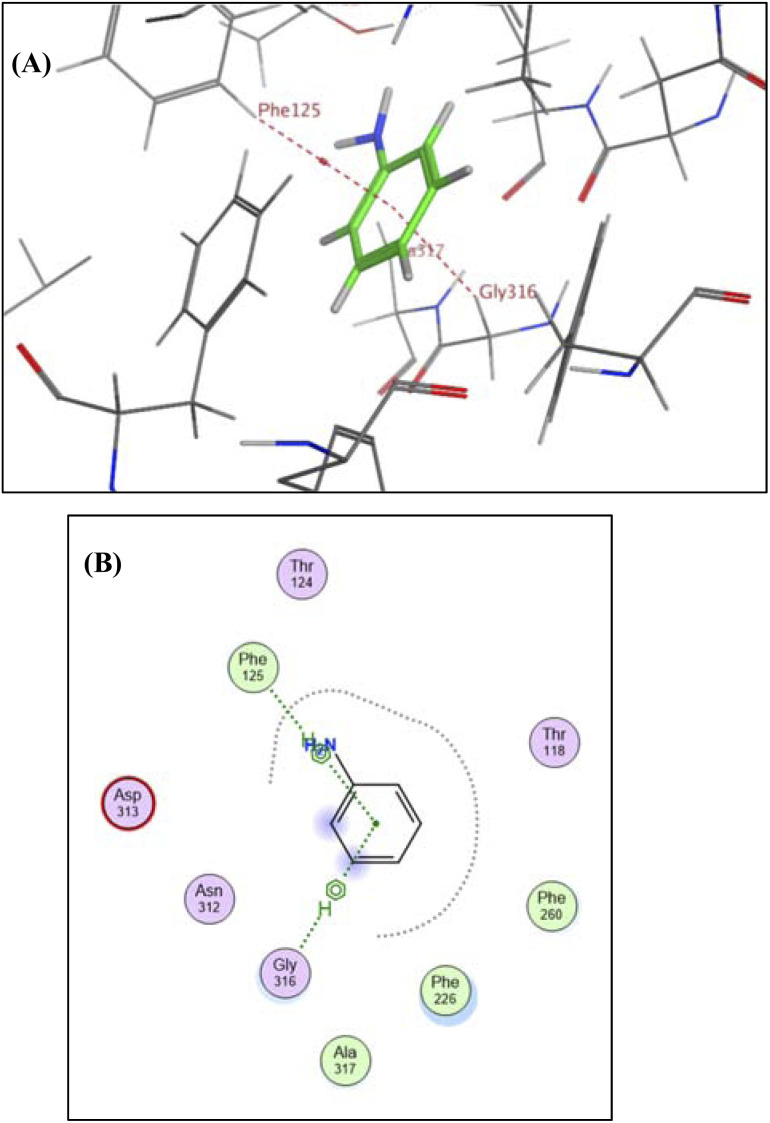
(A) Three-dimensional and (B) two-dimensional molecular simulation of ANN binding with CYP 1A2.

The findings demonstrated that ANN could bind to distinct amino acid residues in the CYP 1A2 compartment either by hydrogen bonding, covalent bond, and electrostatic interaction, as in Phe 125, glycine (Gly 316), and aspartate (Asp 313), respectively. The bond length was 3.00 ± 0.10 Å and its binding energy ranges from 1 to 10 kcal mol^−1^. The binding was demonstrated by 3D and 2D models, respectively (Fig. 5A and B).

Based on previously reported findings, theoretical docking studies verified that ANN had a higher affinity for CYP1A2 and a greater ability to fit.^34^ The validity of the results (Table 7) was assured by the reference sequence (rseq), which represents the primary sequence or the first entry in a list and the model sequence (mseq), which represents alignment or pairing with the reference sequence. Both rseq and mseq results confirmed the appropriate docking process, which released an energy (*S*) of −4.1921.

Validation of the docking protocol is performed *via* calculation of the root mean square deviation (RMSD). The RMSD is predicted *via* redocking the co-crystalized ligand on its target enzyme and then superimposing the redocked co-crystalized ligand onto its original co-crystallized bound conformation. During this study, the RMSD of the Human Microsomal P450 1A2 enzyme was within the acceptable range with a value of 1.2356.

Finally, it is crucial to identify the researched medications in the presence of associated hazardous impurities, such as ANN, to ensure the safety of the pharmaceutical product, as frequent and continuous interaction of ANN with CYP1A2 may cause significant hypoxic injury to human beings.

#### 3.5.2 ADMET LAB 2.0 – drug-likeness and ADME studies

As indicated from ADMET a risk of CNS-related side effects. It did not trigger any structural toxicity alerts for PAINS. Regarding metabolism, ANN exhibited an inhibitory effect on CYP1A2 and CYP2D6 enzymes and acted as a substrate for CYP2C19. In terms of toxicity, it showed a low likelihood of functioning as a hERG blocker, besides the absence of any drug-induced liver injury (DILI).

However, ANN had some safety concerns, a carcinogenicity alert, and a risk of drug-induced respiratory toxicity. In terms of clearance and excretion, ANN displayed a clearance rate of 9.025 mL min^−1^ kg^−1^, which is considered moderate.

### 3.6 Chromatographic method sustainability assessment

**Fig. 6:**
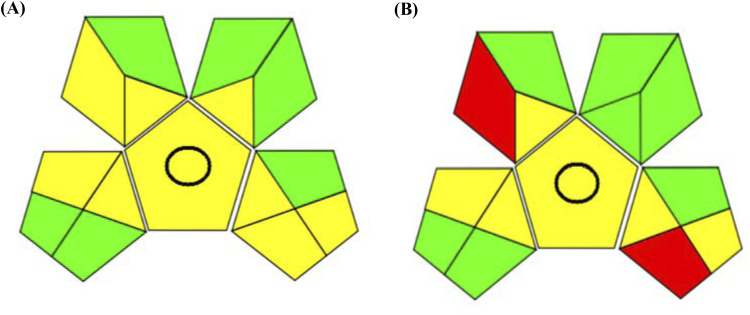
Green assessment using a green analytical procedure index for (A) the proposed and (B) reported HPLC methods.

**Fig. 7:**
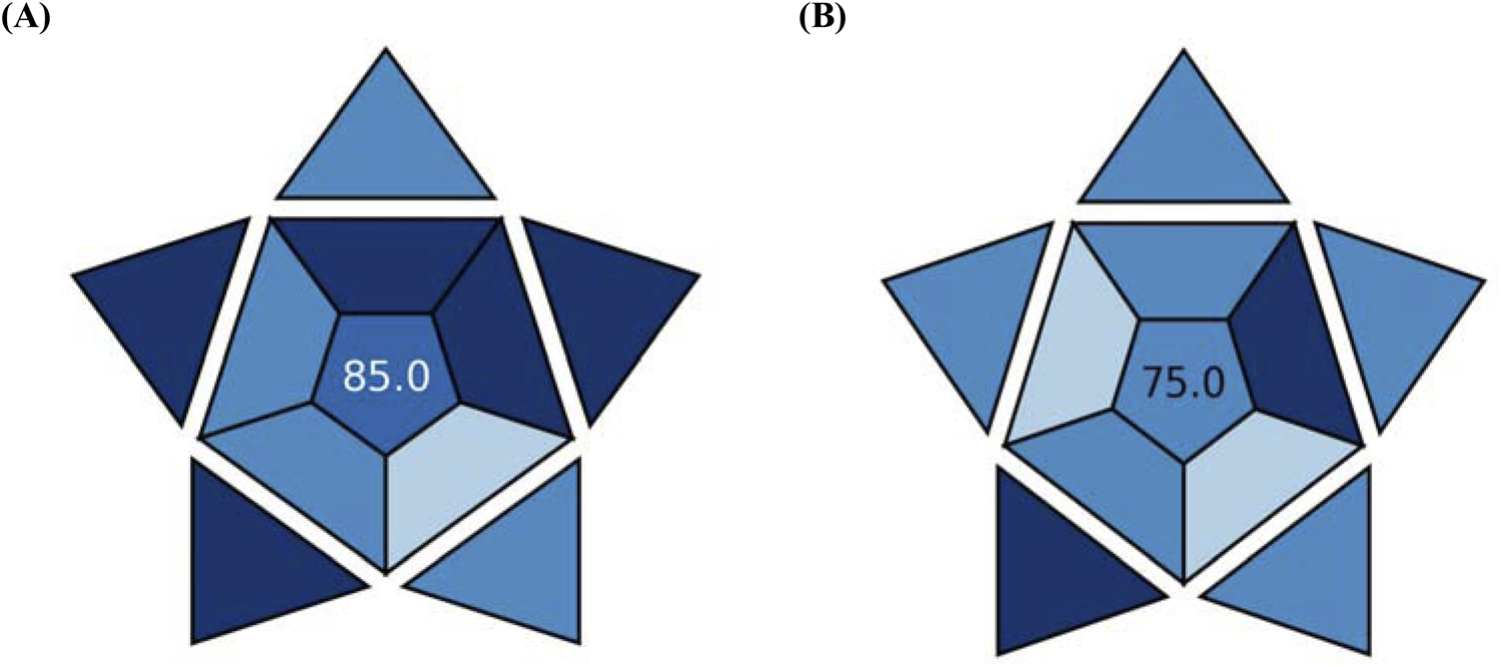
Green assessment using a blue applicability grade index for (A) the proposed and (B) reported HPLC methods.

**Fig. 8:**
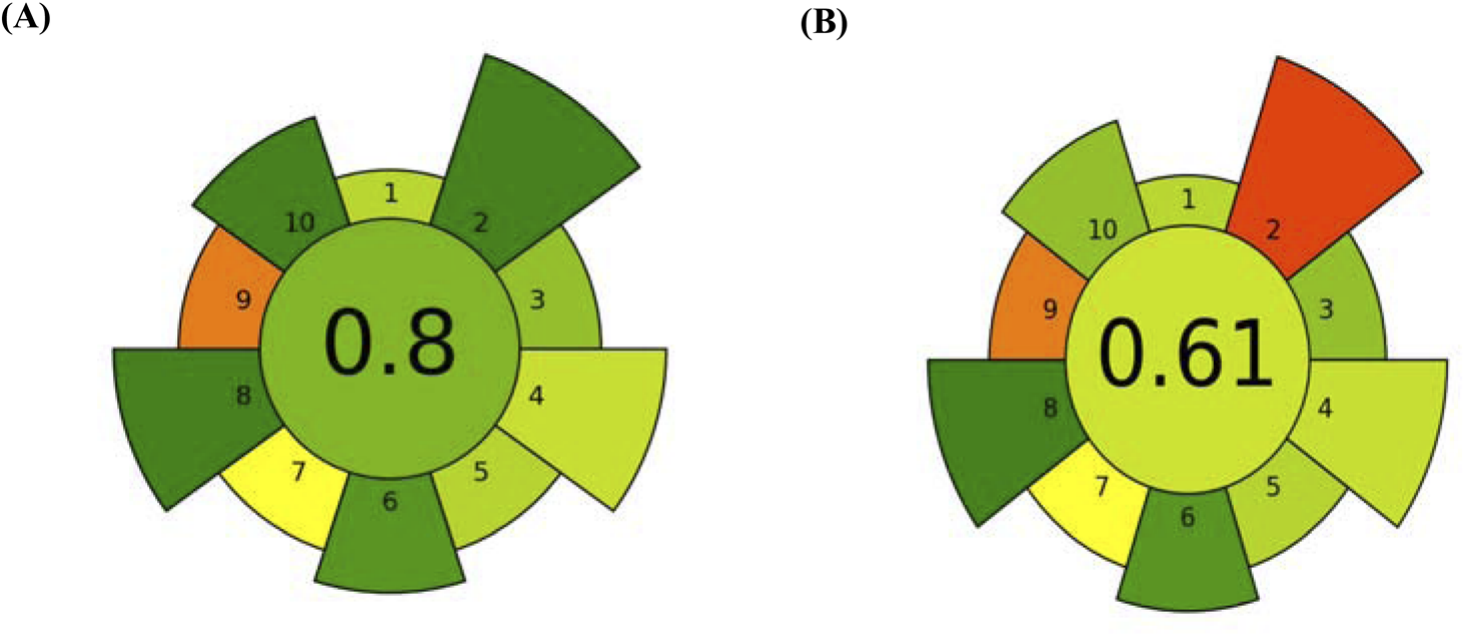
Green assessment using AGREE-Prep for (A) the proposed and (B) reported HPLC methods.

1. The GAPI final assessment is revealed in Fig. 6A, which shows the green assessment for the proposed micellar HPLC method that utilizes SDS and Brij-35 (biodegradable solvents) as key components of the mobile phase. The predominant part of the final GAPI, as shown in Fig. 6A, is green with minimal yellow color, signifying the high level of greenness of the proposed method. Fig. 6B illustrates the GAPI diagram for the reported method,^24^ which revealed superior green sustainability for the proposed method with no red sections, whereas the reported method showed two red sections.

2. BAGI software generated a final graphic showing the degree of greenness (Fig. 7A), illustrating the overall greenness of the proposed method as evaluated by the BAGI system. With a final BAGI score of 85.0, the suggested technique showed excellent applicability. A comparison with the reported method (Fig. 7B)^24^ revealed that the proposed HPLC method is more eco-friendly, as the reported method achieved a lower BAGI score of 75.0.

3. AGREE produced a final estimated greenness score of 0.8 (Fig. 8A) in the middle by allocating different scores to various studied factors. This represents the performance of the greenness approach. The same assessment by AGREE-prep was applied to the reported method^24^ (Fig. 8B).

## 4 Conclusion

Aniline is a toxic degradation product of atorvastatin that may induce methemoglobinemia, a potentially life-threatening condition. Accordingly, its toxicological relevance was investigated using AI-assisted molecular docking and ADMET prediction tools to simulate its interaction with CYP1A2 and to support toxicity assessment. These computational studies were not intended to develop or benchmark new artificial intelligence models; rather, pre-trained AI-based algorithms and scoring functions were employed as supportive predictive and interpretative tools to rationalize the experimental findings. Nevertheless, the proposed computational toxicity assessment is based on predictive models and should be regarded as a preliminary risk-evaluation approach rather than definitive biological confirmation. Therefore, the *in silico* findings are intended to complement, rather than replace, experimental and regulatory toxicological studies. In parallel, a green micellar HPLC method was successfully developed and validated for the simultaneous quantification of aniline and salicylic acid as toxic impurities in aspirin and four widely used cardiovascular drugs, with a short analysis time of less than 10 minutes. The method demonstrated high analytical performance while offering an environmentally sustainable alternative for pharmaceutical analysis. Method greenness was comprehensively evaluated using recent green metrics (GAPI, BAGI, and AGREE), and validation was performed in accordance with ICH guidelines. Overall, the study confirms the toxicological concern associated with aniline and demonstrates the suitability of the proposed eco-friendly micellar HPLC method for its rapid and reliable determination alongside cardiovascular drugs. By integrating AI-assisted toxicity prediction, efficient chromatographic analysis, and green chemistry principles, this work provides a practical and scientifically sound approach to enhance pharmaceutical quality control and support patient safety.

## Author contributions

Alaa Ahmed Mostafa: conceptualization, validation, investigation, writing – original draft. Soad Selem Abd El-Hay: conceptualization, validation, writing – review & editing, supervision. Youstina Mekhail Metias: conceptualization, validation, writing – review & editing, supervision. Mohamed Adel Said: validation, writing – review & editing, software. Michael Gamal Fawzy: conceptualization, validation, writing – review & editing, supervision.

## Conflicts of interest

We have no competing interests to disclose.

## Data Availability

The datasets used and analyzed during the current study are available from the corresponding author on reasonable request.
